# The challenge of treating Roma outpatients in the Eastern Macedonia and Thrace region of Greece

**DOI:** 10.1192/bji.2021.3

**Published:** 2021-08

**Authors:** Theofanis Vorvolakos, Aikaterini Arvaniti, Aspasia Serdari, Maria Samakouri

**Affiliations:** 1 Psychiatrist and Assistant Professor of Psychiatry and Old Age Psychiatry, School of Health Science, Department of Psychiatry, Democritus University of Thrace, Greece, email: tvorvola@med.duth.gr; 2 Psychiatrist and Assistant Professor of Psychiatry, School of Health Science, Department of Psychiatry, Democritus University of Thrace, Greece; 3 Child Psychiatrist Associate and Professor of Child Psychiatry, School of Health Science, Department of Psychiatry, Democritus University of Thrace, Greece; 4 Psychiatrist and Professor of Psychiatry, School of Health Science, Department of Psychiatry, Democritus University of Thrace, Greece

**Keywords:** Roma, Greece, mental health, obstacles, treatment

## Abstract

The Roma people are one of the most unknown and interesting nations in Europe. Although they are severely marginalised within European societies, they have greatly influenced European culture. Despite this fact, there is a deep prejudice against them. In the region of East Macedonia and Thrace, a significant proportion of the population are Roma. Their marginalisation leads to many problems and also affects their mental health. Their psychopathological manifestations differ from the majority population. They express more somatic complaints and higher overall stress in a histrionic background. The main obstacles regarding their mental health issues and treatment appear to be the following: gender inequality, illiteracy and lack of cultural sensitivity in healthcare system. Although all of these obstacles must be removed, some are easier to remove than others. Cultural sensitivity could be applied by using more culturally sensitive diagnostic tools, improving overall training for mental health professionals and treating Roma wherever they seek help, because they often have a nomadic style of living. Telemedicine can be quite useful in serving this goal. Improving their educational status and addressing gender inequalities issues, on the other hand, are more difficult and long-term goals.

The Roma are one of the lesser-known nations in the world. Despite the fact that they live worldwide, there are many issues regarding their origins^1^ and nationality status. Roma people have never had any kind of formal state. They have also never had any written language or formal religion. Their nomadic style of living and separatist attitude lead them to distinguish themselves from the majority population wherever they live.

Despite their differences with majority populations, they have managed to survive for centuries. Their cultural impact on every society in which they have lived, especially those in Europe, is quite notable, especially with respect to music, entertainment and travel.^2^

Unfortunately, their relationship with many Europeans has a very dark side. At present, the Gypsy holocaust, which took place during the Second World War and led to the extermination of at least 400 000 Roma, is widely acknowledged. This is the most striking, but not the only, sign of the deep prejudice that exists, leading to all kinds of discrimination against them. These attitudes toward the Roma often influence the attitude that mental health professionals have toward them, making their diagnosis and treatment more difficult.^3^

The region in which we practice is one of the first regions in which the Roma set foot in Europe. According to many sources, their first appearance in Europe was in the Thrace Region of Constantinople in approximately 1000 A.D., and they have been living in the region since that time. According to the Treaty of Lausanne (1923), a small minority of Muslim Roma were allowed to remain in the Eastern Macedonia and Thrace Region. These individuals account for approximately 8% of the region's population.

## Socioeconomic status and mental health of the Roma population

Almost all of the Roma in the region are Muslims who have settled in a degraded area. Their native language is Turkish. Their religion and language distinguish them from most of the other Roma communities in Greece. They have low incomes and often live in extreme poverty. They express their emotional suffering in different ways in comparison with the majority of the general population, and their overall treatment is quite a challenge for the National Health System.

These diagnosis and treatment difficulties provided the impetus for further study. In the most notable of these studies, we attempted to investigate the course of mental health diseases in the Roma population in the region's out-patient clinic in comparison with the same standard treatment that patients in the majority population were receiving at the same time.

The Roma population was assessed routinely with the Derogatis Psychiatric Rating Scale. This scale is scored by the physician and allows them to record their observations in a more measurable way. A matched majority population was also assessed with the same scale. Before its use, the instrument was translated and standardised in the region, in a mixed population that also included Roma individuals.^4^

Roma patients tend to present more often with increased stress complaints, somatisation, conversion symptoms and agitation, usually with a histrionic and attention-seeking background, when they are suffering from depression or anxiety disorders.^5^ Roma patients respond moderately to treatment, and their progress is slow and unstable; they tend to relapse more often, and their overall stress resists treatment.^6^

## Treatment obstacles

In an attempt to detect any factors that may negatively affect the overall treatment course of Roma individuals, we checked for several such factors, namely, gender, age, marriage status, age of marriage, number of children, social security status, employment, benefits, education, and cultural sensitivity of medical staff. Three of these factors were the main obstacles to improvements in Roma mental health and their treatment response: gender differences, lack of education and lack of cultural sensitivity among health professionals. These statistical research findings concur with our clinical observations. We present our hypothesis for these findings, which are the results of long-standing observations and the treatment of Roma people in the region.

## Gender differences

The percentage of Roma women attending the clinic was, as expected, greater than that of Roma men and that of women in the majority population. Their symptom burden and overall distress were also measured as more severe than those of Roma men.^5^

Women in this population do not enjoy equal status with men. Virginity and endogamy are the two main pillars of their society. As expected, both apply mainly to women. As a result, the mean age of marriage for Roma women in our sample was <17 years, and the most frequent number of children per woman was approximately three. In comparison, for the majority population, the most frequent age at marriage was 21 years, and the most frequent number of children was one. Roma women are practically exposed to the stress of marriage and motherhood stress from their puberty. Furthermore, Islam includes even more guidelines pertaining to gender discrimination.

Such findings are consistent with those of other studies on Roma health status and socioeconomic causality, which identify gender inequality as one of the reasons for the poor health of Roma women.^7,8^

## Lack of education

There are many problems regarding Roma education in the region. First, Roma individuals speak Turkish, so in addition to the fact that they do not have their own written language, this makes it difficult for them to be educated in Greek or Turkish. Furthermore, children often leave school to work or get married. These circumstances lead to very low school attendance: patients attended school for <2 years in comparison with the majority population, who attended school for >8 years. The prevalence of illiteracy among Roma patients is striking, at >85% in comparison with 6% of patients in the majority population. Furthermore, our study found that illiteracy was a strong independent factor that negatively affected the overall outcomes of treatment. One strong hypothesis regarding this finding is that instructions for medication treatment, as well as arrangements for follow-up appointments, are mostly given in writing. Since the illiteracy rate is so high, many Roma tend to be confused regarding their treatment instructions and follow-up appointments.

## Lack of cultural sensitivity

The assessment of psychiatric conditions in the Roma population has been found to be more difficult, since their psychiatric conditions present in a considerably different way than the patients that mental health professionals are accustomed to assessing. It is worth noting that this happens despite there being no such gap in knowledge regarding these issues. The ICD-10, in particular, is sufficiently sensitive to detect syndromes in a specific culture, and the DSM-5 is even more sensitive.^9,10^

Educational courses that raise the cultural sensitivity of mental health professionals toward the Roma people can have a significant impact on professionals’ understanding of their psychopathology and overall treatment.

The treatment framework also creates problems. The main reason for the difference in treatment outcomes or the relapse frequency for the majority population in comparison with Roma patients was the fact that half of the Roma patients have been leaving the treatment framework by no longer attending sessions in out-patient clinics. For the rest of the Roma patients who remained in the treatment framework, however, remission and relapse rates were comparable to those observed in the majority population.^11^

Therefore, instead of expecting them to attend a session in an out-patient clinic, a community mental health team making home visits can be much more effective.

For a nomadic nation such as the Roma, leaving a place is part of their culture. Roma individuals from other areas who move into our region usually seek treatment health problems through our out-patient services. A change in the attitudes of out-patient clinics toward treating them and not referring them back to the services in the places from which they came could be quite useful. Telemedicine services can also contribute tremendously, since medical records can follow every one, and practitioners will also be able to communicate more easily with each other, making it possible for the Roma to move from one place to another without the loss of their continuity of care.^12^

## Conclusions

The treatment of mental health issues in the Roma population is a challenge. Most of the mental health issues and their treatment are related to the insufficient cultural integration of the Roma people, especially regarding women's rights issues and education. A lack of sufficient cultural sensitivity in mental health services also plays an important role.

The achievement of equal status between women and men is a very ambitious and time-consuming aim, and although attempts must be made toward this goal, any results will be very much in the long term. On the other hand, improvement in Roma educational status is an easier goal to achieve, and can help Roma individuals in their integration and, consequently, their mental health status improvement. Adjusting mental health services to their needs is also a much more feasible goal, and can be achieved much sooner.
